# Effect of Taekwondo Practice on Cognitive Function in Adolescents with Attention Deficit Hyperactivity Disorder

**DOI:** 10.3390/ijerph16020204

**Published:** 2019-01-12

**Authors:** Abdelmotaleb Kadri, Maamer Slimani, Nicola Luigi Bragazzi, David Tod, Fairouz Azaiez

**Affiliations:** 1 Higher Institute of Sport and Physical Education of Ksar Said, University of Manouba, Manouba 2010, Tunisia; abdelmottalebkadri@gmail.com; 2 Higher Institute of Sport and Physical Education of Gafsa, University of Gafsa, Gafsa 2100, Tunisia; 3 Postgraduate School of Public Health, Department of Health Sciences (DISSAL), Genoa University, 16132 Genoa, Italy; robertobragazzi@gmail.com; 4 Department of Neuroscience, Rehabilitation, Ophthalmology, Genetics, Maternal and Child Health (DINOGMI), Section of Psychiatry, Genoa University, 16132 Genoa, Italy; 5 School of Sport and Exercise Sciences, Liverpool John Moores University, Liverpool L3 3AF, UK; d.a.tod@ljmu.ac.uk; 6 Higher Institute of Sport and Physical Education of Sfax, University of Sfax, Sfax 3000, Tunisia; fairouz.kyranis@yahoo.com

**Keywords:** ADHD disorder, cognitive functions, taekwondo, martial art

## Abstract

Attention Deficit Hyperactivity Disorder (ADHD) is one of the most common neuro-developmental/behavioral disorders among adolescents. Sport and physical activity seem to play a major role in the development of cognition, memory, selective attention and motor reaction time, especially among adolescents with ADHD. In this context, the objective of this study was to investigate the effects of a one-and-a-half-year-long Taekwondo (TKD) intervention on cognitive function in adolescents with ADHD. Two cognitive instruments, namely the Stroop and the Ruff 2 and 7 tests, were administered to assess attentional inhibitory control and sustained and selective visual attention, respectively. Comparisons between the TKD and control groups at baseline did not reveal significant differences. For post-test scores, there were statistically significant differences on the Stroop color block test (large effect size or ES = 1.26 [95% confidence interval or CI 0.30–2.22]), the color-word interference test (large ES = 2.16 [95% CI 1.10–3.26]), the interference test (large ES = 1.63 [95% CI 0.62–2.64]) and error (large ES = −2.20 [95% CI −3.31 to −1.10]). Similar trends were reported for the Ruff 2 and 7 automated detection trials (large ES = 2.78 [95% CI 1.55–4.01]), controlled search trials (large ES = 2.56 [95% CI 1.38–3.75]) and total speed (large ES = −2.90 [95% CI −4.15 to −1.64]). In conclusion, TKD practice increased selective attention in adolescents with ADHD. Practitioners should implement martial art programs in their general plans to favorably influence attention and health in adolescents with ADHD.

## 1. Introduction

Attention Deficit Hyperactivity Disorder (ADHD) is one the most common neuro-developmental/behavioral disorders among adolescents and young adults [1], with prevalence estimates as wide as 2–18% [2]. ADHD is a complex, chronic mental health disorder, being characterized by: (i) global developmental and learning problems such as autistic spectrum disorders or ASDs, (ii) difficulties with speech and language, motor co-ordination and reading, and (iii) a range of psychiatric disorders as potential co-morbidities.

According to the “Diagnostic and Statistical Manual of Mental Disorders” 5th edition (DSM-V) [1], ADHD can have three clinical presentations: (i) with hyperactivity (restlessness and agitation) and impulsivity (pursuit of immediate reward and loss of critical sense) only, (ii) with inattention (lack of organization and planning) only, or (iii) with both these two types of expression, the so-called combined type [2].

Many causal factors have been implicated in ADHD’s development, including neurological anomalies, hereditary factors, infectious agents, and pre- and post-natal toxic exposures (such as exposure to nicotine, alcohol, carbon monoxide or lead) [3]. The most recent explanations of ADHD have focused on the impairments of the central pre-frontal areas [4]. Generally, scholars believe genetic and environmental factors jointly contribute to ADHD’s development [3].

Young people with ADHD are considered at risk of social isolation and rejection due to poor development of basic motor skills, poor coordination, and low levels of attentional and executive skills [4]. Sport and physical activity, however, seem to play a major role in the development of cognition, memory, selective attention and motor reaction time [5,6,7,8], especially among children with ADHD [9,10]. Potentially, sport and physical activity may help children and adolescents with ADHD. Of note, due to the limited number of studies and the absence of theoretical support provided for the effect of physical activity in adults with ADHD, valid conclusions cannot be drawn.

Within this theoretical framework, taekwondo (TKD) is a Korean martial art, refined over generations, that engages students in a range of cognitive, physical, emotional, social and educational processes [11,12,13,14,15,16,17,18,19,20]. Recently, TKD has received attention from scholars and researchers, who have investigated the benefits of TKD training among children and adolescents, in terms of cognitive and behavioral improvements [21,22,23,24]. However, the possible therapeutic implications of TKD practice still remain overlooked in the extant scholarly literature. For instance, Lakes and Hoyt [21] have compared the effects of TKD training versus the effects of regular physical education in a randomized trial of 207 children aged 5–11 years. Authors showed that school-based TKD training over three months resulted in larger improvements of different functions, including cognitive self-regulation, affective self-regulation, pro-social behavior, classroom conduct, and performance on a mental math test. Cho et al. [22] examined the effects of a 4-month TKD intervention in 30 healthy children. The Color-Word test scores were significantly higher in the TKD group than in the control group after the training period. Lakes and collaborators [25] studied the effects of nine months of TKD training on execution function in youth aged 11–13 years. The TKD group demonstrated significantly greater improvements in a computerized executive function test, which measured accuracy and response time in completing tasks designed to assess inhibitory control, working memory, and cognitive flexibility. In this sense, given the cognitive and social development associated with TKD, examining its influence on adolescents with ADHD may lead to an evidence-based intervention that helps broaden the treatment options for this population. As such, the main objective of this study was to evaluate the effectiveness of a one-and-a-half-year-long TKD training program on cognitive function in young adolescents with ADHD.

## 2. Experimental Section

### 2.1. Population Selection: Inclusion/Exclusion Criteria

The present study is a randomized controlled study registered within the Clinical Trials registry (NCT03678844). Forty young Tunisian patients with ADHD (36 males and four females) were recruited. They were a representative sub-sample of ADHD patients from the Tunis and Sidi Bouzid mental centers (Tunisia). They were randomly allocated to a TKD group (*n* = 20, age = 14.5 ± 3.5 years, 18 males and two females) or a control group (*n* = 20, age = 14.2 ± 3 years, 18 males and two females). The sample size was chosen based on the effect sizes (ES) found in the literature [26] and after a priori power computation, that involved setting the alpha type 1 error at 5%, the beta type 2 error at 20% and allowed capturing an ES ranging from 0.43 to 0.49 [26]. A total sample size (including the control group and the experimental group) of 35–45 members was required. As such, a total sample size of 40 individuals was chosen, who attended all practice sessions. Of note, concerning the TKD group, the participants were of a white belt level pre-intervention. They achieved a blue belt level post-intervention.

To be eligible to participate in the study, participants were required to meet the following criteria: (a) no consumption of any diet supplements or drugs; (b) no history of use of medications that could alter the hypothalamic-pituitary-gonadal (HPG) axis, such as anabolic steroids; (c) no history of chronic disease, bronchospasm or atopy; (d) regular eating patterns; (e) no respiratory infection during the previous month; (f) abstinence from strenuous exercise in the 48 h before testing and (g) not being color blind or vision-impaired.

Local institutional ethical approval was provided for this study, which was conducted in accordance with the 1964 Helsinki declaration and its subsequent amendments. Written informed consent was obtained from the participants and their parents following verbal description of all experimental details, and prior to experimental data collection.

The study was conducted from September 2015 to February 2017. All participants visited the laboratory individually on three separate occasions, at the same time of day (2 pm). The participants were familiarized with the testing procedures at visit 1 and, thereafter, were randomly allocated to one of the two conditions (TKD practice or control condition). The first visit was assigned before 1 week of the intervention and consisted of the collection of anthropometric data. Furthermore, participants were familiarized with the Ruff’s test 2 and 7, as well as with the Stroop test for the assessment of sustained and selective visual attention. During the second visit, participants were asked to perform the above mentioned tests, 1 day before starting one of the two conditions (experimental or control). A rest interval of at least 5-min was provided between tests. The same tests were also performed after each condition during the third visit, 1 day after the last training session. The participants of the TKD group performed various specific TKD techniques and *poomses*. The participants of the control group engaged in physical activities, including athletics, handball and gymnastic, during two sessions of physical education per week at school. Participants were advised to avoid cognitive exercise, and caffeine consumption, 48 h before each laboratory visit. Food and fluid intake was registered 48 h prior to the first study visit, and subjects were asked to avoid such intake 3 h before the second visit.

### 2.2. Measures

In order to assess the impact of TKD practice on cognitive function (namely, two cognitive domains: (i) attentional inhibitory control and (ii) sustained and selective visual attention), two highly utilized, reliable and sensitive instruments to ADHD were administered, namely the Stroop and Ruff 2 & 7 tests [27]. The magnitude of inattention was generally high at the beginning of the study and become lower at the end of the trial.

### 2.3. Stroop Color-Word Test

To assess attentional inhibitory control, the Stroop Color-Word Interference task [28] was performed. Participants were presented with a page consisting of five columns, each with 20 items for a total of 100 items per page. Within each section, the score was computed as the total number of words read aloud in 45 s. Two conditions of this test were applied, namely the Color Block Test and the Color-Word Interference Test. Concerning the Color Block Test, participants were asked to select the appropriate color, as fast as possible, to indicate the names of four colors (red, green, blue, yellow) printed in congruent-color ink (Congruent condition).

Regarding the Color-Word Interference Test, participants were asked to select the appropriate color to name the ink color of words written in a color different from the word’s verbal content, such as the word blue written in red or the word green written in yellow (Incongruent condition). One practice block (10 stimuli) was completed by the participant at the beginning of each condition. The score for each condition was the mean number of correct responses. An interference score was computed by subtracting the mean correct responses for the Congruent condition from the mean correct responses of the Incongruent condition.

### 2.4. Ruff 2 and 7

To evaluate sustained and selective visual attention, we used Ruff’s test 2 and 7 [29]. This test consists of a series of 20 blocks (10 blocks of digits only and 10 blocks of both digits and letters). Each block contained three lines, in each of which 10 targets were interspersed among 40 non-target items. The time to complete each block was limited to 15 s. Participants were required to cross out all instances of the numbers 2 and 7 under two conditions: in the first condition, the targets were distributed among distractors belonging to a different category. This condition relied on a detection using an automatic process of attention. In the second condition, the targets were hidden among distractors belonging to the same category. The automatic and controlled trials scores were computed by calculating the mean of correct responses. The total speed was computed as the total time to complete all blocks within a 5-min time period.

### 2.5. TKD Intervention

The TKD group practiced specific exercises for 50-min twice weekly for a year and a half. Training sessions took place between 3 and 7 pm. The intervention took place in a private martial arts *Dojang*.

The TKD intervention program consisted of the technical skill development aspect of the sport (e.g., blocking, punching and kicking) and *poomse* (forms; they are a series of choreographed physical movements performed with technical precision in a particular order). This second activity consists of different stages in which high levels of attention are needed to execute the movements, including blocks and kicks, with precision in the correct order. At each stage of progression (recognized by moving to higher levels that are noted by different belt colors), movements become more challenging (increasing both the number of steps or actions and the complexity and physical difficulty of the behaviors). TKD students were expected to be able to perform all previously learned forms as well as new forms, for 30-min. Before and after each training session, participants completed a 10-min general warm-up (stretching, jogging and strengthening, sitting-up and pushing-up) and recovery, respectively. The TKD intervention was conducted by qualified TKD instructors. Of note, no other physical activity was performed by participants post-intervention.

### 2.6. Statistical Analysis

Concerning the statistical analysis, data were visually inspected for potential outliers, before proceeding with their manipulation. They were represented as group mean values and standard deviations (SD). Range values were also reported where appropriate. After verifying the data normality assumption with the Shapiro-Wilk test (which was preferred to other normality tests due to the small sample size used in the present investigation), exploratory independent Student’s t-tests were used to detect baseline differences between the groups and training effects over time, using Bonferroni correction to ensure protection against multiple testing. Dependent variables were analyzed using separate 2 (Groups: TKD group—control group) × 2 (Time: pre, post) analysis of variance (ANOVA).

ES were determined by calculating Cohen’s *d* values along with their 95% confidence interval or CI [30]. Cohen’s *d* values were classified as small (0.00 ≤ *d* ≤ 0.49), medium (0.50 ≤ *d* ≤ 0.79), and large effects (*d* ≥ 0.80) according to the Cohen’s rule of thumb [30]. Global ES for mean differences between the two groups taking into account the pre-post design were computed according to Morris [31].

Furthermore, a pre-post difference score was computed for all the variables studied and regression models were run.

A priori power analysis computation of the sample size was carried out with the software G*Power version 3.1.9.2 (Heinrich-Heine-Universität Düsseldorf, Düsseldorf, Germany). A significance level of *p*-value ≤ 0.05 was set for all analyses other that the t-tests where the Bonferroni adjustment was applied. All statistical analyses were carried out using the commercial software Statistical Package for the Social Sciences for Windows version 16.0 (SPSS Inc., Chicago, IL, USA).

## 3. Results

Table 1 presents the mean and SD results for the dependent variables. Comparisons between the TKD group and the control group on pre-test measures did not reveal any statistically significant differences in attention performance. For post-test scores, there were statistically significant differences between the groups on the Stroop color block test (large ES = 1.26 [95% CI 0.30–2.22], *p* < 0.001), the color-word interference test (large ES = 2.16 [95% CI 1.10–3.26], *p* < 0.001), the interference test (large ES = 1.63 [95% CI 0.62–2.64], *p* < 0.001) and error (large ES = −2.20 [95% CI −3.31 to −1.10], *p* < 0.001). Similar trends were reported for the Ruff 2 and 7automated detection trials (large ES = 2.78 [95% CI 1.55–4.01], *p* < 0.001), controlled search trials (large ES = 2.56 [95% CI 1.38–3.75], *p* < 0.001) and total speed trials (large ES = −2.90 [95% CI −4.15 to −1.64], *p* < 0.001). Higher scores were reported for the Stroop test (except for error) and the Ruff 2 and 7 test (except for the total speed trials) in the TKD condition than in the control condition, which indicates the beneficial effect of TKD practice on these abilities. Concerning the Ruff 2 and 7 controlled search trials, the effects of the time and of the group were statistically significant (F = 39.99, *p* < 0.001, and F = 32.26, *p* < 0.001, respectively), as well as the interaction of time and group (F = 68.93, *p* < 0.001). Concerning the automatic detection trials, the effects of the time and of the group were significant (F = 39.99, *p* < 0.001 and F = 32.26, *p* < 0.001, respectively) as well as the interaction of time and group (F = 68.93, *p* < 0.001). Concerning the total speed trials, the effects of the time and of the group were statistically significant (F = 43.07, *p* < 0.001 and F = 41.03, *p* < 0.001, respectively), as well as the interaction of time and group resulted significant (F = 74.34, *p* < 0.001). Finally, the regression models were statistically significant for all the variables under scrutiny: namely, for automatic detection trials (regression coefficient = 51.60, standard error or SE = 4.27, r_partial_ = 0.89, t = 12.10, *p* < 0.0001), for controlled search trials (regression coefficient = 49.60, SE = 5.04, r_partial_ = 0.85 t = 9.83, *p* < 0.0001) and for total speed trials (regression coefficient = −54.85, SE = 3.72, r_partial_ = −0.92, t = −14.76, *p* < 0.0001).

For the Stroop color test, group (F = 4.75, *p* = 0.032), time (F = 3.97, *p* = 0.050) and group X time interaction (F = 18.94, *p* < 0.001) effects were statistically significant. Similar trends were obtained for the Stroop word test for all the effects under study—namely, group (F = 10.09, *p* = 0.002), time (F = 10.09, *p* = 0.002) and group X time interaction (F = 40.36, *p* < 0.001) effects. For the Stroop word-color test, the effects of group (F = 13.56, *p* < 0.001) and of time (F = 10.28, *p* = 0.002) were statistically significant, as well as the interaction of time and group (F = 39.43, *p* < 0.001). For the Stroop error, both the effects of time (F = 5.78, *p* = 0.019) and of the group (F = 16.42, *p* < 0.001), as well as the interaction of time and group (F = 26.54, *p* < 0.001) resulted in significant differences. Concerning the Stroop interference, the effect of time was borderline significant (F = 2.97, *p* = 0.089), whereas the effects of the group (F = 4.43, *p* = 0.039) and of the interaction of time and group (F = 17.31, *p* < 0.001) resulted in statistically significant differences. Finally, the regression models were statistically significant for all the variables investigated: namely, for the Stroop color test (regression coefficient = 25.45, SE = 3.78, r_partial_ = 0.74, t = 6.73, *p* < 0.0001), for the Stroop word test (regression coefficient = 28.40, SE = 1.51, r_partial_ = 0.95, t = 18.79, *p* < 0.0001), for the Stroop word-color test (regression coefficient = 23.70, SE = 2.10, r_partial_ = 0.88, t = 11.28, *p* < 0.0001), for the Stroop error (regression coefficient = −3.75, SE = 0.52, r_partial_ = −0.76, t = −7.25, *p* < 0.0001) and for the Stroop interference (regression coefficient = 8.76, SE = 1.47, r_partial_ = 0.69, t = 5.95, *p* < 0.0001).

## 4. Discussion

To the best of our knowledge, this is the first study to evaluate the effectiveness of a long-term TKD training intervention on cognitive function in adolescents with ADHD. The findings showed that the participants who received the TKD program had a better cognitive performance in terms of selective attention than those in the control condition after the training program.

Concerning ADHD, in the literature, the majority of studies have involved cognitive rehabilitation to improve cognitive function [32,33,34]. These studies have reported that cognitive interventions, namely a computerized progressive attentional training program and a Cogmed Working Memory Training Program, can enhance attention, working memory and non-verbal reasoning ability, and may reduce behavioral symptoms of ADHD in children [32,33,34]. This improvement may be explained by the fact that cognitive training increases brain activity in the parietal and frontal regions linked to working memory [35] and altered dopamine D1 receptor binding in both the prefrontal and parietal cortices [36].

In addition to cognitive training, some studies have reported that physical training improves cognitive function in children and adolescents with ADHD [37,38]. This enhancement may be due to the improvement of hippocampal long-term potentiation (LTP) [39], neurogenesis [40,41], hippocampal and neocortical neurotrophin mRNA expression [41,42], cerebellar blood vessel density [43], a blunted catecholamine response [44], changes in eye blink responses and reductions in motor impersistence [45].

Amongst physical activities, previous investigations have suggested that the martial arts are one avenue to mental and physical health [46,47]. More specifically, it has been shown that both Tai Chi, as a traditional Chinese martial art, and TKD reduce anxiety, daydreaming behaviors, inappropriate emotions, and hyperactivity in adolescents with ADHD [21,25,48,49].

In extending current knowledge, the present study’s novel findings report that the TKD group outperformed the control group on all the cognitive development dependent variables: Stroop color, word-color, interference time and Ruff 2 and 7 test performance in terms of automatic detection trials, controlled search trials and total speed at post-test, with no differences at the pre-test. These findings may reflect that TKD training required greater attention demands than did standard physical education. Thus, it is worth noting that TKD is a very sophisticated activity which meshes both physical and mental components and leads to the balance and harmony of the body, mind and spirit [21]. This aspect leads to increased levels of attention in adolescents with ADHD. In addition, it has been shown that TKD training may increase brain activity and functional connectivity which explained the improvement of cognitive function [35,36]. The current findings extend the results of previous studies that revealed significant increases in Stroop Color and Word Test scores following regular TKD training in healthy children [21,22,49]. These findings are also in line with the results of previous meta-analyses which examined the effect of physical training on cognitive function in children with and without ADHD. Cerrillo-Urbina et al. [50] reviewed 18 randomized controlled trials that examined the effect of aerobic and yoga exercise on attention in children with ADHD. They reported that aerobic training had a moderate effect on attention (ES = 0.84). For instance, the ES reported in the previous meta-analysis was slightly lower than that observed in the present study (ES = 1.26–2.9). Fedewa and Ahn [51] examined the effect of physical activity on cognitive outcomes in children. The authors reported that aerobic training had significantly greater differences on cognitive outcomes than either general physical education or perceptual motor training.

## 5. Strengths and Limitations

Our study has some strengths and limitations that should be recognized. The main strengths include the experimental randomized design with a control group and the long intervention period. Several instruments have been used to collect information and descriptive and experimental data. Concerning major limitations, the small sample size used for the present study consisted mostly of males and it is difficult to generalize the findings more widely. Future studies are needed to replicate our findings and extend them in other populations.

## 6. Conclusions

Taking into account the above-mentioned limitations, the present study showed that TKD practice may increase selective attention of adolescents with ADHD, in terms of the Stroop color block test, the color-word interference test, the interference test, the Ruff 2 and 7 automated detection trials and controlled search trials. Consequently, TKD could be considered as an appropriate non-pharmacological therapeutic method to combat/counteract the attentional impairment of individuals with ADHD from a younger age. Practitioners should implement martial art programs in their school-based physical education classes to favorably influence attention and for the treatment of ADHD. However, due to the shortcomings of the present investigations, further high-quality research in the field is urgently needed.

## Figures and Tables

**Table 1:** The impact of Taekwondo (TKD) practice on cognitive attention tests in the experimental and control groups.

Variables	TKD Group			Control Group			TKD vs. Control Group at Baseline	Global Effect Size
	Pre	Post	Statistical Significance	Pre	Post	Statistical Significance		
Stroop test								
Color Block Test	57.4 ± 9.3 (39–70)	75.9 ± 18.2 (7–93)	<0.001	63.7 ± 11.5 (35–80)	56.8 ± 11.4 (39–77)	<0.001	0.063	2.41
Color-Word Interference Test	41.0 ± 7.7 (29–57)	58.9 ± 5.9 (50–73)	<0.001	45.9 ± 8.4 (31–61)	40.1 ± 10.8 (7–53)	<0.001	0.064	2.92
Word Test	76.5 ± 8.5 (60–91)	97.8 ± 10.8 (80–120)	<0.001	83.6 ± 11.1 (60–102)	76.5 ± 9.5 (55–91)	<0.001	0.028	2.85
Interference	8.3 ± 5.1 (−1.2–19.2)	14.5 ± 4.0 (8.8–20.9)	<0.001	10.5 ± 5.3 (2.7–21.6)	7.9 ± 4.1 (0.9–15.1)	0.003	0.201	1.68
Error	4.0 ± 2.0 (1–8)	1.3 ± 0.7 (0–3)	<0.001	3.6 ± 1.5 (1–7)	4.6 ± 2.0 (2–8)	0.007	0.479	−2.08
Ruff 2 and 7 test								
Automatic detection trials (correct responses)	138.2 ± 12.2 (107–152)	183.6 ± 18.9 (155–220)	<0.001	146.3 ± 11.7 (116–163)	140.2 ± 11.4 (110–155)	<0.001	0.037	4.28
Controlled search trials (correct responses)	110.3 ± 11.4 (83–124)	154.2 ± 18.9 (123–190)	<0.001	120.0 ± 19.1 (94–132)	114.4 ± 11.2 (89–131)	0.011	0.005	3.13
Total speed trials (seconds)	288.6 ± 11.6 (253–300)	240.3 ± 19.7 (197–267)	<0.001	281.6 ± 11.3 (246–293)	288.1 ± 12.5 (251–300)	<0.001	0.059	−4.75

Mean values and standard deviations (SD) of the attention tests (the Stroop and the Ruff 2 and 7 tests) pre- and post-intervention, their statistical significance and their global effect sizes (ES).
